# C-type natriuretic peptide/guanylyl cyclase-B (GC-B) system attenuates proliferation of rhabdomyosarcoma in combination with sildenafil: A novel anticancer therapy

**DOI:** 10.1186/2050-6511-16-S1-A103

**Published:** 2015-09-02

**Authors:** Masahiro Zenitani, Shuichiro Uehara, Toru Kimura, Takashi Nojiri, Koichi Miura, Hiroshi Hosoda, Mikiya Miyazato, Hiroomi Okuyama, Kenji Kangawa

**Affiliations:** 1 Department of Biochemistry, National Cerebral and Cardiovascular Center Research Institute, Suita-City, Osaka, Japan; 2 Department of Pediatric Surgery, Osaka University Graduate School of Medicine, Suita-City, Osaka, Japan; 3 Department of Regenerative Medicine and Tissue Engineering, National Cerebral and Cardiovascular Center Research Institute, Suita-City, Osaka, Japan

## Background/purpose

Rhabdomyosarcoma (RMS) is the most common soft tissue sarcoma in children. Since patients with high-risk RMS have limited treatment options and a poor prognosis, and late complications including growth disturbance and secondary cancer induced by chemotherapy or radiation therapy are essential problems, novel therapies are urgently needed. C-type natriuretic peptide (CNP) has physiological activity mainly for mesenchymal cells to inhibit cell proliferation. We hypothesized that CNP attenuates proliferation of human RMS, a malignant mesenchymal tumor and provide rationale for using CNP to treat RMS.

## Experimental design

Gene expression of guanylyl cyclase B (GC-B), receptor of the CNP in rhabdomyosarcoma clinical samples and cell lines was assessed by using quantitative polymerase chain reaction. To ascertain biological activity of CNP in RMS cells, cyclic guanosine monophosphate (cGMP), the second messenger molecule which is produced by the activation of GC-B and serves as the major signaling molecule was measured following addition of CNP. However, cGMP is quickly degraded by phosphodiesterases and thereby biological activity becomes deactivated. Therefore, sildenafil, a selective inhibitor of phosphodiesterase 5 (PDE5) was added to CNP to prolong or enhance the effects of physiological processes mediated by cGMP by inhibiting its degradation. The in vitro and in vivo efficacy of CNP and sildenafil was evaluated alone and in combination.

## Results

GC-B expression was seen in all alveolar and embryonal clinical samples which had higher GC-B expression than RMS cell lines. Since RD and RMS-YM cell lines had considerably lower GC-B expression than the clinical samples and KYM-1 and RH30 had no detectable expression, GC-B stable expression cell lines, RD-GC-B was established to mimic the clinical samples. CNP increased the rate of cGMP accumulation in RMS cells depending on concentration of this peptide. Combinations of CNP and sildenafil synergistically inhibited cell growth of RD-GC-B cells by inhibiting extracellular-signal-regulated kinase (Erk), the major signal for cell proliferation in RMS. The synergistic effect on growth and reduction of phosphorylated Erk as tumor pharmacodynamic biomarker were also confirmed in a mouse xenograft model.

## Conclusions

These results suggest that CNP in combination with sildenafil may have clinical efficacy for treating RMS patients.

